# Methylphenidate attenuates the response to cold pain but not to aversive auditory stimuli in healthy human: a double-blind randomized controlled study

**DOI:** 10.1097/PR9.0000000000000593

**Published:** 2017-04-14

**Authors:** Dorit Pud, Eelena Broitman, Omar Hameed, Erica Suzan, Joshua Aviram, May Haddad, Salim Hadad, Rafi Shemesh, Elon Eisenberg

**Affiliations:** a Department of Nursing, Faculty of Social Welfare and Health Sciences, University of Haifa, Haifa, Israel; b Department of Communication Sciences and Disorders, Faculty of Social Welfare and Health Sciences, University of Haifa, Haifa, Israel; c The Rappaport Faculty of Medicine, Technion—Israel Institute of Technology, Haifa, Israel; d Institute of Pain Medicine, Rambam Health Care Campus, Haifa, Israel; e Department of Pharmacy, Rambam Health Care Campus, Haifa, Israel

**Keywords:** Experimental pain, Dopamine, Norepinephrine, Pain threshold, Pain tolerance, Methylphenidate

## Abstract

**Introduction:**

We recently showed that the psycho-stimulant norepinephrine–dopamine reuptake inhibitor methylphenidate (MP) prolonged cold pain threshold and tolerance in adults with attention-deficit hyperactivity disorder (ADHD).

**Objectives:**

The objectives of the present study were to: (1) examine whether MP has antinociceptive properties in healthy men; (2) test MP's effects on responses to aversive auditory stimuli. The underlying aim was to determine whether MP exerts antinociceptive properties or more generalized, nonspecific attenuating effects on different aversive sensory modalities.

**Methods:**

This double-blind, crossover, randomized placebo-controlled study consisted of 2 sessions one week apart from each other. In each session, pain threshold (seconds) and tolerance (seconds) in response to painful cold stimulation were measured. Additionally, threshold (dB) and tolerance (seconds) to loud aversive auditory stimuli (500 Hz, 2000 Hz and white noise) were also tested prior to and 2 hours following the administration of a single dose of either 20 mg MP or an identical looking placebo.

**Results:**

Forty men, 26.1 ± 4.0 (mean ± SD) years were enrolled in the study. Wilcoxon signed-rank test analyses showed that MP, but not the placebo, produced a significant increase in cold pain threshold (from 4.1 ± 2.6 to 5.4 ± 3.1 seconds, *P* = 0.001 and from 4.5 ± 2.6 to 4.3 ± 2.7 seconds, *P* = 0.2, respectively) and tolerance (from 57.8 ± 54.0 to 73.8 ± 61.8 seconds, *P* = 0.001 and from 52.5 ± 53.7 sec to 57.0 ± 52.9 seconds, *P* = 0.1, respectively). No significant changes were found in any of the auditory parameters.

**Conclusion:**

These results suggest that MP has an effect on nociceptive pathways rather than a nonspecific, generalized attenuating effect on aversive sensory stimuli.

## Abstract 2

This double-blind, crossover, randomized placebo-controlled study found that methylphenidate has a specific effect on nociceptive pathways rather than a generalized effect on aversive sensory modalities.

## 1. Introduction

The psycho-stimulant, methylphenidate (MP, Ritalin) is the most commonly used treatment for attention-deficit hyperactivity disorder (ADHD).^8,29,35^ It is a central nervous system stimulant and produces clinical effects such as increasing or maintaining alertness, combating fatigue, and improving attention.

Although MP has not become a part of the pharmacological armamentarium in pain medicine, some evidence suggests that it may have analgesic properties. Johnstone (1974)^23^ showed that MP relieved postoperative pain and Cantello et al (1988)^9^ reported that MP exerted an analgesic effect in a group of patients with Parkinson's disease. In one case study by Mellick and Mellick (1998),^30^ refractory cluster headache was relieved by MP. In contrast, a double-blind study failed to demonstrate an effect of MP on postoperative pain in comparison with a placebo.^14^ It should be noted though that all these reports are primarily case studies and small-scale trials therefore provide only limited evidence for MP efficacy.

Recently, we have shown in an open-label study that MP prolonged both the latency of onset and the tolerance to experimental cold pain in a cohort of ADHD adults.^37^ Based on these results, the present study aimed to explore the analgesic properties of MP in healthy subjects exposed to experimentally evoked cold pain, in a randomized, controlled fashion. Assuming that such an analgesic effect would indeed be demonstrated, the second aim of this study was to examine if this effect is nociceptive specific or is part of a nonspecific effect of MP on different aversive sensory brain functions. Hence, the effect of MP on aversive auditory stimuli was tested as well. The auditory modality was chosen because, like cold stimuli, it has a nonaversive and aversive range; its aversive range can be evaluated and quantified in a psychophysical manner (ie, threshold and tolerance) and in that range it can be applied safely without causing harm.^25^

## 2. Methods

### 2.1. Subjects

The study included 40 healthy men. Only men were recruited due to sex differences in the response to painful stimuli. The minimum sample size for this within-subject and between-subjects repeated-measures design was calculated by G* Power analysis^16,28^ to include 34 subjects (power [1 − β] = 0.8; α = 0.05; effect size = 0.4). Subjects were eligible for enrollment in the study if they were healthy and free from pain of any type, reported normal hearing, declined the use of any medications or other substances, were able to understand the purpose and instructions of the study, and did not meet the DSM-V criteria for ADHD.^1^ Subjects were excluded from the study if hearing loss was diagnosed at the beginning of the first experimental session (see below). The study was approved by the institutional ethics committees at Rambam Health Care Campus (#0275-13-RMB) and at the University of Haifa (#065-13).

### 2.2. Pain tests

Pain tests were conducted at the human experimental pain research laboratory at the University of Haifa by one experimenter (E.B.).

#### 2.2.1. Cold pain threshold and tolerance

The cold pressor test (CPT) apparatus^26^ was used for cold pain measurements (Heto CBN 8-30 Lab equipment, Allerod, Denmark). The CPT is a temperature-controlled water bath with a maximum temperature variance of ±0.5°C, which is continuously stirred by a pump. Subjects were asked to place their right hand in the CPT (1°C) in a still position with their fingers spread wide apart. A stopwatch was simultaneously activated, and the subjects were requested to maintain their hand in the cold water for as long as they could. They were instructed to indicate the exact point in time when the cold sensation began to elicit pain. This time point of the first perception of pain was defined as the cold pain threshold, measured in seconds. The latency to spontaneous hand removal was defined as the pain tolerance, measured in seconds. A cutoff time of 180 seconds was set for safety reasons. The pain tolerance for subjects who did not withdraw their hand for the entire 180 seconds was recorded as 180 seconds.

### 2.3. Auditory tests

Hearing tests were conducted in an acoustic test chamber at the Interdisciplinary Clinical Center University of Haifa, by one experimenter (O.H.). All tests were performed in the right ear only, in order to reduce differences between the 2 ears responses (similar to the pain tests in the right hand).

#### 2.3.1. Hearing threshold (audiogram)

For the detection of possible hearing loss, hearing thresholds were tested. Subjects were exposed to gradually increased intensities (by up5/down10 dB steps) of pure tones through headphones (Telephonics TDH 39). Using audiometer model: AC40, Interacoustics, (Middelfart, Denmark), the following frequencies were administered: 250, 500, 1000, 2000, 4000, 8000 Hz, and white noise. Subjects were instructed to press a button every time they heard a sound.^2^ Normal hearing was considered if sounds were detected below 20 dB for all frequencies.^22^

#### 2.3.2. Aversive auditory threshold

For the purpose of this study, we defined the threshold for loud “aversive auditory perception” as a sound intensity that was perceived as aversive at its onset. Subjects were instructed that they would hear gradually increasing sounds and were requested to press a button as soon as the sound became aversive. Subjects were then exposed to increasing intensities (5 dB steps) of sounds at 500 Hz, 2000 Hz and white noise. The cutoff point was set to 100 dB. Pressing the button immediately stopped all sounds. The threshold was measured in dB.

#### 2.3.3. Aversive auditory tolerance

To evaluate the tolerance to aversively loud sounds, subjects were instructed to release a pressed button at the moment they could no longer tolerate the loud sound (500 Hz, 2000 Hz and white noise). The intensities of these sounds were those perceived as the aversive auditory thresholds (see above), but did not exceed either 100 dB or 15 minutes (according to National Institute for Occupational Safety, NIOSH).^31^ Tolerance was measured in seconds.

### 2.4. Study medications

Methylphenidate sustained-release (Ritalin SR) 20 mg tablets (the lowest available dose of SR tablets) or lactose powder (placebo) were inserted into opaque capsules by Rambam Health Care Campus pharmacy. Thus, a single dose of MP or identical looking placebo capsules were administered orally to all subjects in a cross-over fashion. This formulation was selected to assure that the duration of action of MP would cover the entire test period (http://www.medsafe.govt.nz/profs/datasheet/r/RitalintabSRtabLAtab.pdf). Randomization of the order of MP and placebo administration was done in blocks of 4 according to a computer-generated random code. Randomization and blinding were done by an investigator who had no contact with the tested subjects.

### 2.5. Study design

The study was designed as a double-blind, crossover, randomized, placebo-controlled trial. Subjects were recruited by responding to advertisements at the University of Haifa. Subjects were informed that they are requested to participate in a study examining the influence of MP or placebo on the sensitivity to pain and hearing. However, no information was provided on the expected effect of the study drugs on the outcomes. Subsequent to completing a screening telephone questionnaire, eligible subjects were requested to complete the inventory for excluding ADHD^1^ and were then invited for the first experimental session that consisted of a detailed explanation of the study procedure, the signing of written informed consent, and a baseline hearing test. The study included 2 sessions that were conducted at the same time of the day at the University of Haifa, one week apart from each other. Each session lasted approximately 6 hours. Both hearing and cold-pain trainings were administered 10 minutes prior to the beginning of the actual experiments in the 2 sessions in order to familiarize the subjects with the tests. In each session, subjects were exposed to baseline experimental pain and auditory tests: pain 1, auditory 1 (or vice versa). They then received one tablet of either MP or a placebo in randomized order (ie, MP in the first session and placebo in the second session or vice versa). Right after, subjects were asked stay in a rest room near the laboratory and 2 hours later, they were exposed to the same experimental pain and auditory stimuli: pain 2, auditory 2 (or vice versa). A 10-minute interval between 2 consecutive tests was allowed. The time frame of the study was based on published data on clinical effects of Ritalin SR (http://www.medsafe.govt.nz/profs/datasheet/r/RitalintabSRtabLAtab.pdf).

**Figure 1.**
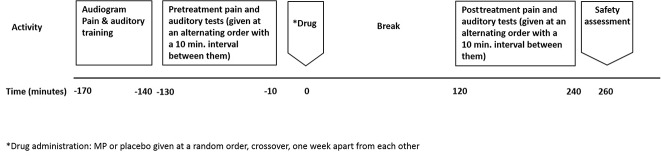
Study design.

The timing of the interventions and measurements is depicted in Figure 1.

### 2.6. Adverse events

At the end of each session, subjects were given a list of common MP-related adverse effects and were requested to report if they had experienced any of them and at what level of severity (mild, moderate, or severe).

### 2.7. Statistical analyses

All analyses were conducted using the SPSS for Windows version 20 statistical package (SPSS, Inc, Chicago, IL). Descriptive statistics were generated for all pain and auditory outcomes. A Shapiro–Wilk W test of normality (Analyse-it, version 2.20; Analyse-it Software Ltd, Leeds, United Kingdom) revealed that all tested measures were not normally distributed; hence, all analyses were based on nonparametric tests. Wilcoxon signed-rank test analyses were computed in order to identify significant differences between pretreatment and posttreatment values of the tested measures, separately for MP and for the placebo. Results were considered significant at the 0.05 level. Results are presented as mean (SD), median (interquartile range, IQR), and minimum to maximum values.

## 3. Results

Forty-three men were assessed for eligibility to participate in the study. Forty-two of them (aged 26.1 ± 4.0 [mean ± SD] years [ranging from 20–37 years]) met the inclusion criteria (one had Asthma). Yet, since 2 subjects declined participation after enrollment, only forty subjects completed the study.

### 3.1. Pain tests

Comparing between the 2 baselines pain tests (pre-MP vs preplacebo) revealed no significant differences in the 2 tested parameters. Specifically, the mean ± SD cold pain thresholds were 4.1 ± 2.6 (median 3.5, IQR 3.7) vs 4.6 ± 2.6 (median 4.0, IQR 2.0) sec pre-MP and preplacebo, respectively (Z = −1.9; *P* = 0.06), and the cold pain tolerances were 57.8 ± 54.5 (median 31.0, IQR 55.0) vs 52.5 ± 53.6 (median 29.0, IQR 40.0) sec (Z = −0.80; *P* = 0.42).

**Figure 2.**
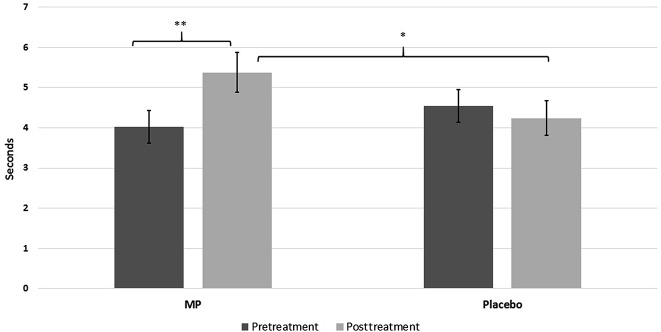
Cold pain threshold (in seconds) prior to (black bars) and following (grey bars) MP and placebo administration. *P < 0.05; **P = 0.001.

**Figure 3.**
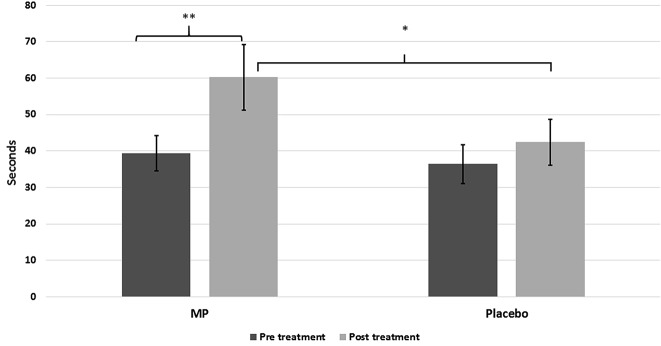
Cold pain tolerance (in seconds) prior to (black bars) and following (grey bars) MP and placebo administration. *P < 0.05; **P = 0.001.

The differences between pretreatment and posttreatment were significant for both cold pain threshold and tolerance in response to MP but not to the placebo. Following MP, cold pain threshold increased from 4.1 ± 2.6 (median 3.5, IQR 3.7) seconds to 5.4 ± 3.1 (median 4.6, IQR 4.0) seconds (Wilcoxon test, Z = −3.3; *P* = 0.001, Table 1 and Fig. 2) and cold pain tolerance increased from 57.8 ± 54.0 (median 30.5, IQR 55.0) seconds to 73.8 ± 61.8 (median 41, IQR 103.0) seconds (Wilcoxon test, Z = −3.22; *P* = 0.001, Table 1 and Fig. 3).

**Table 1:**
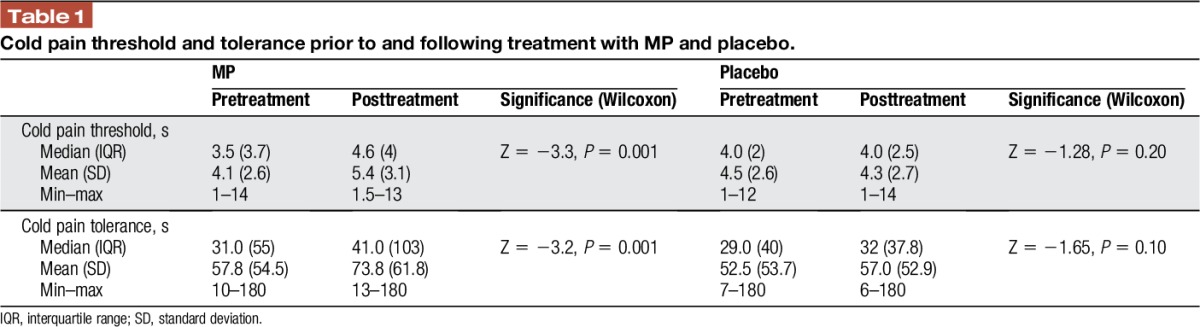
Cold pain threshold and tolerance prior to and following treatment with MP and placebo.

At the posttreatment testing, significant differences between treatments were found for both cold pain threshold and tolerance (Wilcoxon test, Z = −2.704, *P* = 0.007, Fig. 2, Z = −2.212, *P* = 0.027, Fig. 3, respectively).

### 3.2. Auditory tests

The baseline (pre-MP and preplacebo) auditory tests for aversive auditory thresholds and tolerance did not differ from each other at any of the 3 tested frequencies. The mean baseline aversive auditory thresholds were in the range of 96 to 97 dB (median 100, IQR 5–9) and the mean baseline tolerance ranged from 38 to 66 seconds (median 12.5–35, IQR 20–88), depending on the tested frequencies (Tables 2 and 3). Importantly, the aversive auditory stimuli were not perceived as painful by any of the subjects.

**Table 2:**
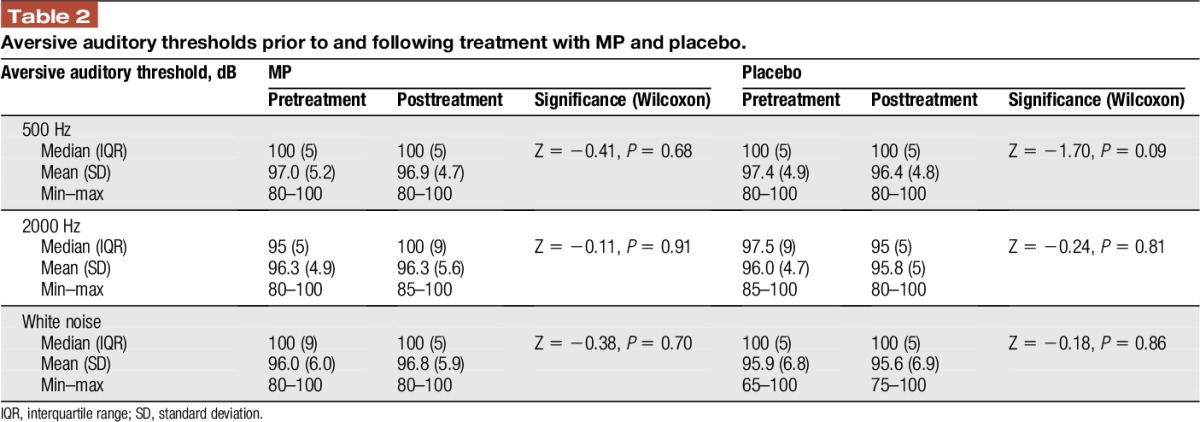
Aversive auditory thresholds prior to and following treatment with MP and placebo.

**Table 3:**
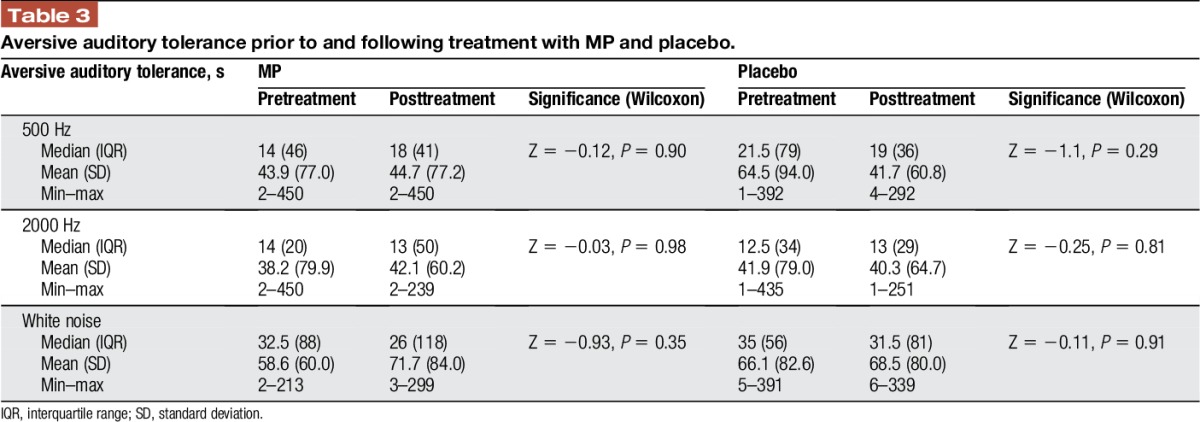
Aversive auditory tolerance prior to and following treatment with MP and placebo.

In addition, no significant changes were found between pretreatment and posttreatment (ie, MP and placebo) in the tested auditory parameters at any of the 3 tested frequencies (Tables 2 and 3).

### 3.3. Adverse events

No adverse events were reported by any of the subjects.

## 4. Discussion

The present study demonstrated under placebo-controlled conditions that MP had a prolonging effect on the latency and tolerance to experimental cold pain in healthy subjects. At the same time, MP had no effect on any of the corresponding aversive auditory outcome measures. Although recent evidence shows that an aversive tissue-damaging noise may elicit a nociceptive response, termed “auditory nociception,”^17^ the aversive auditory stimuli did not cause pain in any of the tested subjects. We therefore believe that these results suggest that MP may have an effect on nociception rather than a generalized attenuating effect on aversive sensory modalities.

Most previous studies on MP and pain have focused on MP's pain-reducing properties in different painful conditions.^9,14,23,30^ The results of these studies, even when positive, have demonstrated only limited pain-reducing effects of MP when administered alone with no other therapy. Interestingly, from a slightly different perspective, Bruera et al (1987)^6^ has shown an opioid potentiating analgesic effect of MP in patients with cancer pain. In a recent study, we have shown that MP may have a different analgesic effect; rather than reducing pain intensity, it prolonged threshold and tolerance to cold pain in a sample of adults with ADHD.^37^ The results of the present study are in congruence with that observation since they show again a prolonging effect of MP on the threshold and tolerance to cold pain in healthy subjects under controlled conditions.

Methylphenidate is a central nervous system stimulant that primarily increases extracellular dopamine levels by blocking the dopamine transporter.^27,40^ It augments dopamine efflux in all major dopaminergic nerve terminal regions of the brain, leading to prolonged dopamine receptor interactions.^27^ Methylphenidate also acts as a norepinephrine reuptake inhibitor. It binds and blocks norepinephrine transporters although to a lesser extent than it blocks dopamine transporters.^20,21^

A possible explanation for the tolerance enhancing effect of MP is the involvement of dopamine in the reward systems. Dopamine has been shown to influence motivation and willingness to continue enduring an intense stimulus.^5^ However, in that case, one could expect MP to prolong not only the tolerance to cold pain but also to the aversive auditory stimuli. Thus, this explanation seems fairly unlikely.

Could it then be a specific dopaminergic analgesic effect? A number of pharmacological trials have suggested that medications with dopaminergic activity can reduce pain in patients with conditions that are linked to abnormalities in dopaminergic neurotransmission, such as Parkinson's disease,^4,18^ fibromyalgia,^42^ and restless leg syndrome.^10^ Other clinical reports have shown that dopamine can reduce pain in patients with conditions which are not associated with dopaminergic abnormalities, such as bone metastases,^13,32^ painful diabetic neuropathy,^16^ and herpes zoster.^24^ Additionally, studies from our laboratory have demonstrated genetic associations between dopamine-related gene polymorphisms and cold pain tolerance,^39^ and a prolonging effect of the dopamine agonist apomorphine on cold pain tolerance in healthy subjects.^38^ In contrast, a recent study by Becker et al^3^ failed to show an analgesic effect of dopaminergic manipulations on various forms of experimental pain in healthy subjects, including thermal pain threshold and tolerance. Therefore, currently, there is no firm concept regarding the exact role of the dopaminergic system in pain processing and further studies are clearly warranted.^34,41^

An alternative explanation is related to the fact that MP does not act purely on the dopaminergic system. As mentioned earlier, it also has norepinephrine reuptake inhibiting properties,^20,21^ which are known to enhance descending pain inhibition. Numerous studies have demonstrated analgesic effects of antidepressant agents with norepinephrine (and serotonin) reuptake inhibitory properties.^11^ However, very little is known about their effect on experimental cold pain. We have been able to identify 2 studies in which a single dose of imipramine (a strong serotonin and norepinephrine reuptake inhibitor) had no effect on cold pain tolerance^15,35^; although imipramine is not a pure noradrenergic compound, these findings raise questions regarding to the contribution of norepinephrine to the MP prolonging effect on cold pain tolerance.

The tolerance-prolonging effect of MP can potentially have an encouraging clinical perspective. Due to the limited effectiveness of currently available treatments in reducing the intensity of many chronic painful conditions, developing other strategies for pain management is clearly required.^19^ Thus, interventions aimed at enhancing pain tolerance and facilitating functioning—despite the presence of unrelieved pain—can be advantageous. In line with this, MP, which seems to increase ability to tolerate pain, could possibly be useful for achieving this goal. Additionally, the interactions between opioid and dopaminergic systems, which may lead to analgesic synergism, suppression of morphine-induced rewarding effects and reduction of opioid induced sedation, are all of potential clinical interest.^7,12,33^

Four limitations of the study should be noted. First, skin temperature of the hand was not measured prior to its immersion in the cold water. Theoretically, MP could have produced peripheral autonomic effects such as skin temperature changes,^36^ leading in turn to alteration in the perception of the cold stimuli. Although we have not been able to find any evidence for such an effect in relation to MP, this possibility cannot be ruled out. Therefore, in future studies involving drugs with potential peripheral autonomic effect and experimental thermal pain models, measuring baseline skin temperature should be considered. Second, subjects were not asked at the end of each session whether or not they could identify the active drug from the placebo. Although no side effects were reported, such identification could have contributed to the reliability of the drug-blinding procedure. Third, it should be noted that the mean baseline aversive auditory thresholds were in the range of 96 to 97 dB. This is in close proximity to the preselected cutoff point, which was set to 100 dB. This proximity might have limited the ability to detect any drug effect of the aversive auditory threshold. The lack of effect of the experimental drug on the aversive auditory threshold should therefore be interpreted cautiously. One additional point deserves consideration is the lack of placebo effect on the outcome measures. This may reflect the fact that no indication on the expected effect of MP on their pain experience (ie, reducing or enhancing) was given to the participants. Thus, no expectation was built. This may also explain, at least in part, the absence of adverse events. Forth, only men were recruited to the present study, thus limiting generalizability of the findings to females.

In summary, the results suggest that MP has an enhancing effect on thresholds and tolerance to experimental cold pain in healthy subjects. This effect seems to be antinociceptive rather than a generalized attenuating effect on aversive sensory modalities. Further studies aimed at determining the utility of these properties of MP in the clinical practice are warranted.

## Disclosures

The authors state no conflict of interests regarding this work.

Supported by unrestricted research grants from Novartis Pharma Services AG, Israel, and from the Israel Pain Association.
